# COL8A1 as a pro-inflammatory mediator bridges immune evasion and therapy resistance in glioma

**DOI:** 10.3389/fimmu.2025.1727298

**Published:** 2025-12-10

**Authors:** Jiachong Wang, Jiale Li, Jun Peng, Chunyuan Zhang, Zigui Chen, Changfeng Miao, Chunhai Tang, Qisheng Luo

**Affiliations:** 1 Department of Neurosurgery, Haikou Affiliated Hospital of Central South University Xiangya School of Medicine, Haikou, China; 2 Department of Neurosurgery, Affiliated Hospital of Youjiang Medical University for Nationalities, Baise, Guangxi, China; 3 Guangxi Engineering Research Center for Biomaterials in Bone and Joint Degenerative Diseases, Baise, Guangxi, China; 4 Department of Neurosurgery Second Branche, Hunan Provincial People’s Hospital (The First Affiliated Hospital of Hunan Normal University), Changsha, Hunan, China; 5 Department of Neurosurgery, The Second Affiliated Hospital of Guangxi Medical University, Nanning, Guangxi, China

**Keywords:** glioma, COL8A1, single-cell sequencing analysis, immunotherapy, inflammation

## Abstract

**Background:**

Glioma remains the most aggressive and therapy-resistant brain tumor, with a highly immunosuppressive tumor microenvironment. The role of inflammatory signaling in glioma progression and treatment response is poorly understood.

**Methods:**

We performed single-cell RNA sequencing (scRNA-seq) analysis on 93,027 cells from 18 samples. Inflammation-related genes were identified using hdWGCNA and AUCell scoring. Multiple bulk RNA-seq and microarray datasets were integrated for validation. Machine learning algorithms, including CoxBoost, LASSO, and Random Survival Forest, were used to identify prognostic genes. Immune infiltration, immunotherapy response, and mutational landscape were analyzed using established computational tools.

**Findings:**

COL8A1 was found to be a significant prognostic gene within a highly linked gene module connected to inflammation. Astrocytes, OPCs, and cancerous cells all had high levels of COL8A1 expression. In several cohorts, low survival was linked to high COL8A1 expression. The suppression of tumor migration and proliferation by COL8A1 knockdown was validated by functional tests. Multiple immunotherapy determinants, inhibitory immunological checkpoints, and immune cell infiltration all showed high correlations with COL8A1 expression. Additionally, it accurately forecasted the immune checkpoint blockade response. Through mutational profiling, we identified distinct somatic mutation patterns distinguishing COL8A1-high from COL8A1-low cancers.

**Conclusion:**

By connecting tumor-intrinsic inflammation to immunological surveillance and treatment resistance, our study identified COL8A1 as a crucial inflammatory hub in glioma. In order to improve the results of immunotherapy for glioma, COL8A1 may be a useful therapeutic target and prognostic biomarker.

## Introduction

Glioma represents the most common and lethal primary intracranial malignancy. Despite decades of research and the implementation of multimodal standard-of-care treatment, comprising maximal safe surgical resection, concomitant radiotherapy and temozolomide chemotherapy, and adjuvant temozolomide, the median survival of glioblastoma (GBM) patients remains a dismal 12 to 15 months (1). The profound intra- and inter-tumoral heterogeneity, coupled with an intensely immunosuppressive tumor microenvironment (TME), constitutes a formidable barrier to therapeutic success. The recent advent of immunotherapy, which has revolutionized the treatment landscape for numerous solid malignancies, has yielded largely disappointing results in GBM clinical trials (2). This underscores the urgent need to deepen our understanding of the intricate cellular and molecular interactions within the glioma TME that underpin immune evasion and therapy resistance.

Inflammation is a hallmark of cancer that exerts a dualistic influence on tumorigenesis and progression (3). On one hand, chronic inflammation can foster a pro-tumorigenic milieu by promoting angiogenesis, tissue invasion, and suppression of anti-tumor immunity (4). However, acute inflammatory reactions might enhance the activation and recruitment of cytotoxic immune cells, which can help destroy tumors. A varied cellular consortium comprising resident microglia, bone marrow-derived macrophages, lymphocytes, and the tumor cells themselves, which frequently hijack inflammatory signaling pathways for survival and growth, shapes the inflammatory landscape in glioma, which is especially complex (5, 6). However, the specific drivers of these inflammatory programs and their precise impact on the anti-tumor immune cycle and ultimate patient outcomes are not fully delineated.

The collagen family, traditionally viewed as structural components of the extracellular matrix (ECM), has recently gained attention for their multifaceted roles in tumor biology (7). Collagen type VIII alpha 1 chain (COL8A1), a short-chain collagen, is involved in various physiological and pathological processes, including angiogenesis and tissue remodeling (8). Aberrant expression of COL8A1 has been reported in several cancers (9); however, its role in glioma, particularly its connection to the inflammatory TME and immune regulation, remains largely unexplored.

The emergence of high-throughput sequencing technologies, especially single-cell RNA sequencing (scRNA-seq), provides an unprecedented opportunity to deconvolute the cellular heterogeneity of glioma and dissect gene expression programs at a single-cell resolution (10). This allows for the identification of critical molecular hubs that operate within specific cell populations and govern broader TME states.

In this work, we mapped the inflammatory landscape at cellular resolution using a large-scale scRNA-seq dataset. Through the integration of network biology and machine learning techniques across several bulk transcriptome cohorts, our goal was to find and validate important genes that connect inflammation to immune surveillance and tumor growth. In glioma, our research methodically identified COL8A1 as a unique inflammatory center. We provide a convincing case for targeting COL8A1 to enhance glioma therapy by thoroughly characterizing its prognostic value, validating its pro-tumorigenic functions *in vitro*, unraveling its complex role in forming an immunosuppressive TME, assessing its predictive power for immunotherapy response, and examining its association with various mutational landscapes.

## Materials and methods

### Dataset collection and processing

The scRNA-seq dataset GSE174554, comprising 18 tumor samples, was analyzed (11). The R package Seurat was used to process the 93,027 cells in the scRNA-seq dataset (12). Following global-scaling normalization and logarithmic transformation, we selected highly variable genes. The Harmony was used to address cross-sample heterogeneity (13). UMAP was employed to visualize the cellular distribution. Cell clustering was conducted by applying the Louvain method to a shared nearest-neighbor graph, and the resulting clusters were annotated based on the identification of marker genes and comparison with established canonical markers.

Glioma bulk RNA sequencing datasets were collected, including TCGA cohort (14), CGGA311 cohort (15), CGGA668 cohort (15), GSE108474 cohort (16), GSE13041 cohort (17), GSE83300 cohort (18), GSE4271 cohort (19), GSE4412 cohort (20), and GSE68838 cohort. Microarray data from Affymetrix platforms were processed using the affy R package, with background adjustment, quantile normalization, and log2 transformation applied via the RMA algorithm. For bulk RNA-seq data, expression values were normalized to FPKM and then converted to TPM. Due to the comparable dynamic ranges between RMA-adjusted microarray data and TPM-standardized RNA-seq, we proceeded with cross-platform integration for further comparative analyses.

### Computational analysis

We obtained a list of inflammation-related genes from HALLMARK pathways (21). For scRNA-seq data, inflammation levels in cancer cells were measured using the AUCell method (22). We then performed high-dimensional gene network analysis (hdWGCNA) on the cancer cells to find gene modules most linked to inflammation (23). Genes expressed in fewer than 5% of cells were excluded to prevent technical noise from confounding biological signals. To mitigate single-cell data sparsity, cells were aggregated into metacells, each representing transcriptomic profiles of approximately 25 cells. Strict limits on shared cells between any two metacells ensured their statistical independence, avoiding artifactual correlations. A soft power threshold of ten was then selected to create a scale-free co-expression network. Using ConsensusClusterPlus (24) with the PAM algorithm, we identified inflammation-based cancer subgroups (24). The limma package helped detect differences in gene expression between these subgroups (25). We analyzed genes from the key module (blue module) using univariate Cox regression to assess their impact on patient survival. Additional analyses, including Random Survival Forest (26), LASSO (27), and CoxBoost (28), helped refine the list of predictive genes. We evaluated immune system activity using the cancer immune cycle framework (29) and measured immune cell infiltration with MCPcounter (30), Porpimol’s study (31), and TIMER (32). We also examined how COL8A1 interacts with seven types of immune regulators (33). To predict COL8A1’s role in immunotherapy response, we analyzed nine established markers, including CYT (34), IFNγIS (35), RohIS (36), DavoliIS (32), chemokineIS (37), RIR (38), ICBnetIS (39), AyersExpIS (35), and GEP (35). We used KEGG pathway analysis and Metascape to understand COL8A1’s biological functions (40). The maftools was utilized to generate the mutation landscape (41).

### *In vitro* validation

To validate the functional role of COL8A1, we performed *in vitro* knockdown using two specific siRNAs in U251 and LN229 GBM cells, with knockdown efficiency confirmed by RT-qPCR. The U251 and LN229 cell lines were purchased from the Cellverse Co., Ltd. Functional assays included EdU incorporation to assess cell proliferation and Transwell assays to evaluate cell migration. Additionally, we employed a coculture system to examine the paracrine effect on microglia, where the migratory capacity of HMC3 microglial cells was measured after treatment with conditioned medium from COL8A1−silenced or control GBM cells. The HMC3 cell line was also purchased from the Cellverse Co., Ltd. The U251, LN229, and HMC3 cell lines were all authenticated by STR profiling. U251 and LN229 cells were cultured in Dulbecco’s Modified Eagle Medium (DMEM, Gibco) supplemented with 10% fetal bovine serum (FBS, Gibco) and 1% penicillin/streptomycin (Gibco). HMC3 cells were cultured in Minimum Essential Medium (MEM, Gibco) supplemented with 10% FBS and 1% penicillin/streptomycin. All cells were maintained in a humidified incubator at 37 °C with 5% CO_2_. For COL8A1 knockdown, two specific siRNAs and a negative control siRNA (NC) were designed and synthesized. The target sequences for COL8A1-siRNA1 and COL8A1-siRNA2 were 5’-GCCACCACAAATTCCACAATA-3’ and 5’-GCCTATGAGATGCCTGCATTT-3’, respectively. Transfection was performed using Lipofectamine 3000 (Invitrogen) according to the manufacturer’s protocol when cells reached 60-70% confluence. Functional assays (EdU and Transwell) were conducted 48 hours post-transfection. For detailed procedures of these experiments, please refer to our previous study (42).

## Results

### Deciphering the inflammation-associated gene network in glioma at single-cell resolution

**Figure 1:**
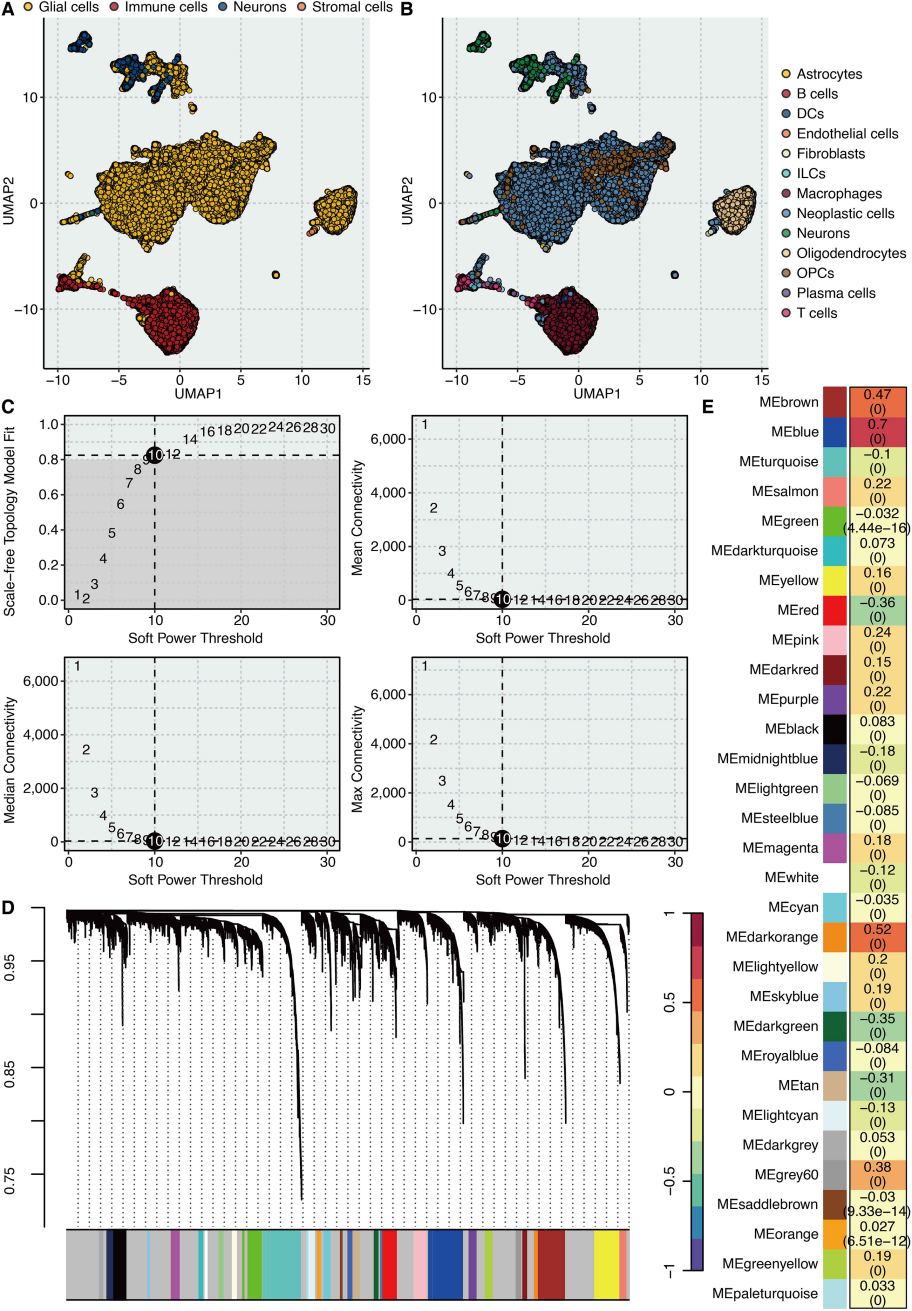
Identification of “Inflammation”-related genes in glioma at the scRNA-seq level. **(A)** UMAP shows the major cell types in the tumor microenvironment of glioma. **(B)** UMAP shows the minor cell types in the tumor microenvironment of glioma. **(C)** The connection between soft power threshold and scale-free topology model fit, mean connectivity, median connectivity, and max connectivity under the hdWGCNA circumstance. **(D)** The Waterfall plot shows the distribution of gene models in cancer cells. **(E)** The correlation between the “Inflammation” feature and gene models.

To unravel the inflammatory interplay within the glioma TME, we began by analyzing the scRNA-seq dataset, which encompasses 93,027 cells from 18 tumors. UMAP effectively visualized the major cellular constituents of the TME, broadly categorized into glial, immune, neuronal, and stromal cells (**Figure 1A**). Further annotation resolved specific cell subtypes, including B cells, T cells, dendritic cells (DCs), endothelial cells, macrophages, oligodendrocyte precursor cells (OPCs), plasma cells, and astrocytes, providing a detailed cellular census of glioma (**Figure 1B**).

We then focused our analysis on the malignant cell population, as they are the primary architects of the TME. Using the AUCell method, we quantified an “Inflammation” activity score for each cancer cell based on the HALLMARK inflammatory gene signature. To identify co-regulated gene sets associated with this inflammatory state, we performed hdWGCNA on the cancer cells. The scale-free topology fit analysis determined an optimal soft power threshold of 10 for network construction (**Figure 1C**). hdWGCNA successfully partitioned the highly variable genes into several distinct co-expression modules, each represented by a unique color (**Figure 1D**). Crucially, correlation analysis between module eigengenes and the “Inflammation” score revealed that the blue module exhibited the strongest positive correlation (correlation = 0.7, p < 0.001), highlighting it as the most significant inflammation-related gene network in cancer cells (**Figure 1E**).

### Stratification of glioma by inflammation signature and identification of COL8A1 as a core prognostic determinant

**Figure 2:**
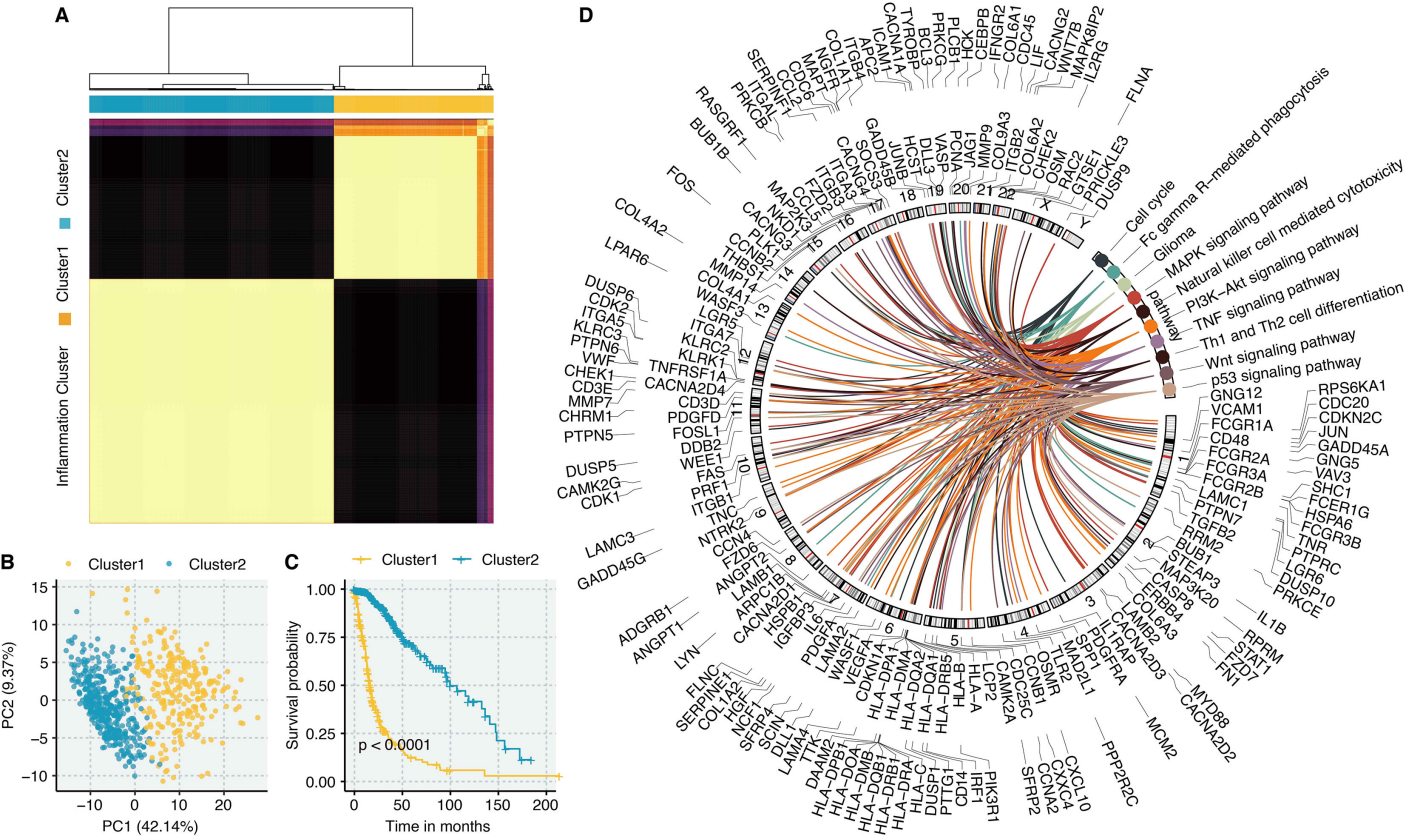
Development of “Inflammation”-related clusters in glioma. **(A)** The matrix of PAM-based clusters at the k-value of two. **(B)** PCA plot shows the sample distribution in two clusters. **(C)** Survival curves show the survival outcomes of two clusters in the TCGA cohort. **(D)** The distribution of DEGs between two clusters in human chromosomes.

To translate our single-cell findings to a bulk tissue context and assess clinical relevance, we utilized the genes from the blue module to cluster glioma samples in the TCGA cohort using the Partitioning Around Medoids (PAM) algorithm. At k=2, the algorithm robustly segregated patients into two distinct clusters (Cluster 1 and Cluster 2, **Figure 2A**), which were clearly separated in principal component analysis (PCA) space (**Figure 2B**). Strikingly, survival analysis revealed that patients in Cluster 1 suffered from significantly shorter overall survival compared to those in Cluster 2 (**Figure 2C**), establishing the clinical prognostic value of this inflammation-related gene set. We identified differentially expressed genes (DEGs) between the two clusters and visualized their distribution across the human genome (**Figure 2D**).

**Figure 3:**
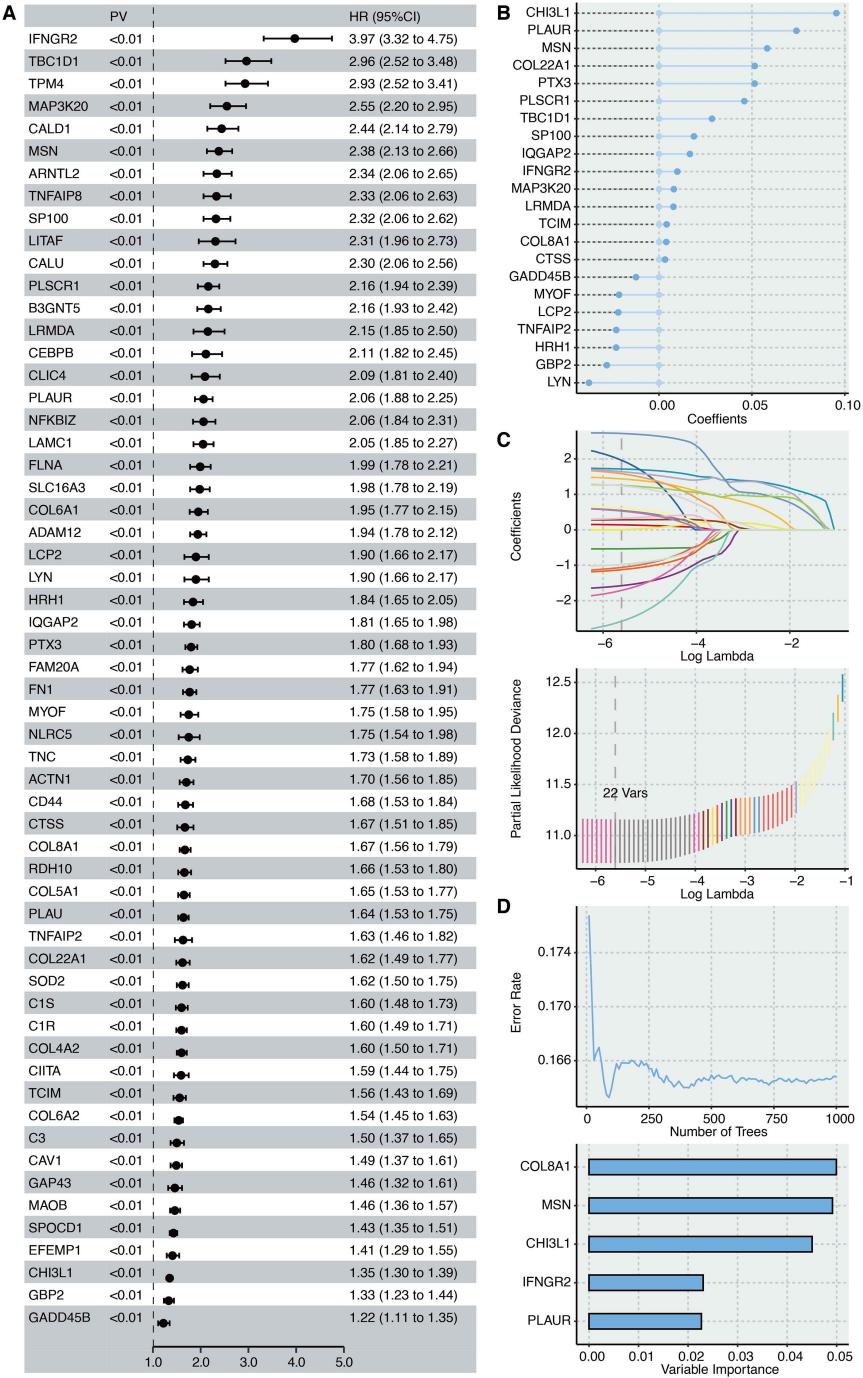
Identification of COL8A1 as a protective marker in glioma. **(A)** Univariate Cox regression analysis shows the prognostic genes in the combined pink, midnightblue, and purple module genes. **(B)** CoxBoost shows the feature genes after dimension reduction of prognostic genes. **(C)** LASSO shows the feature genes after dimension reduction of prognostic genes. **(D)** Random Forest shows the feature genes after dimension reduction of prognostic genes.

To pinpoint the most critical driver genes, we intersected three gene lists: 1) genes from the hdWGCNA blue module, 2) genes upregulated in tumor cells compared to non-malignant cells, and 3) DEGs between the two inflammation-based clusters. This tripartite intersection yielded 66 high-confidence candidate genes (**Supplementary Figure S1A**). To refine this list to the most potent prognostic factors, we performed univariate Cox regression analysis on the intersected genes, identifying those with significant associations with overall survival (**Figure 3A**).

Subsequently, we employed three distinct machine learning algorithms for dimensionality reduction and feature selection (43–45). Both CoxBoost (**Figure 3B**) and LASSO regression (**Figure 3C**) independently narrowed the list down to 22 key prognostic genes. The Random Survival Forest algorithm, which assesses variable importance, further prioritized the top 5 genes (**Figure 3D**). Notably, COL8A1 consistently emerged as the most significant gene across all analyses, ranking highest in variable importance in the Random Forest model, thereby identifying it as the most potent prognostic marker within the inflammation network.

**Figure 4:**
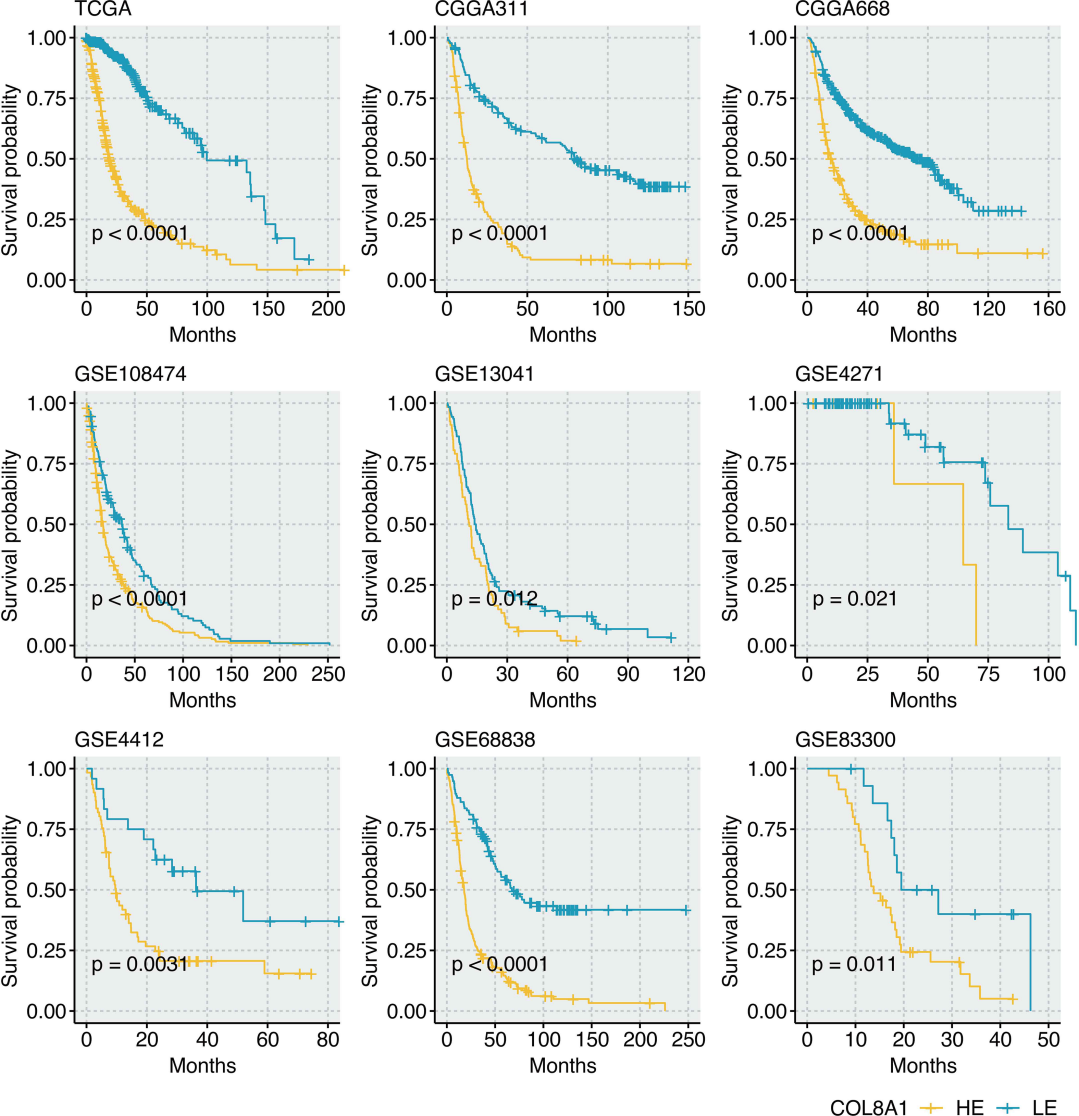
Prognostic value of COL8A1 in glioma. A. Survival curves show the survival outcomes of COL8A1-based groups in the TCGA cohort, CGGA311 cohort, CGGA668 cohort, GSE108474 cohort, GSE13041 cohort, GSE4271 cohort, GSE4412 cohort, GSE68838 cohort, and GSE83300 cohort.

Examination of COL8A1 expression at the single-cell level revealed its predominant expression in neoplastic cells, with additional expression in OPCs and astrocytes, suggesting both tumor-intrinsic and stromal sources contribute to its levels in the TME (**Supplementary Figure S1B**). The prognostic power of COL8A1 was then rigorously validated across nine independent bulk transcriptomic cohorts (TCGA, CGGA311, CGGA668, GSE108474, GSE13041, GSE4271, GSE4412, GSE68838, GSE83300). In every cohort, patients with high COL8A1 expression exhibited significantly worse overall survival, unequivocally establishing COL8A1 as a robust and reproducible poor prognostic factor in glioma (**Figure 4**).

### *In vitro* functional validation of COL8A1 in promoting glioma aggressiveness

**Figure 5:**
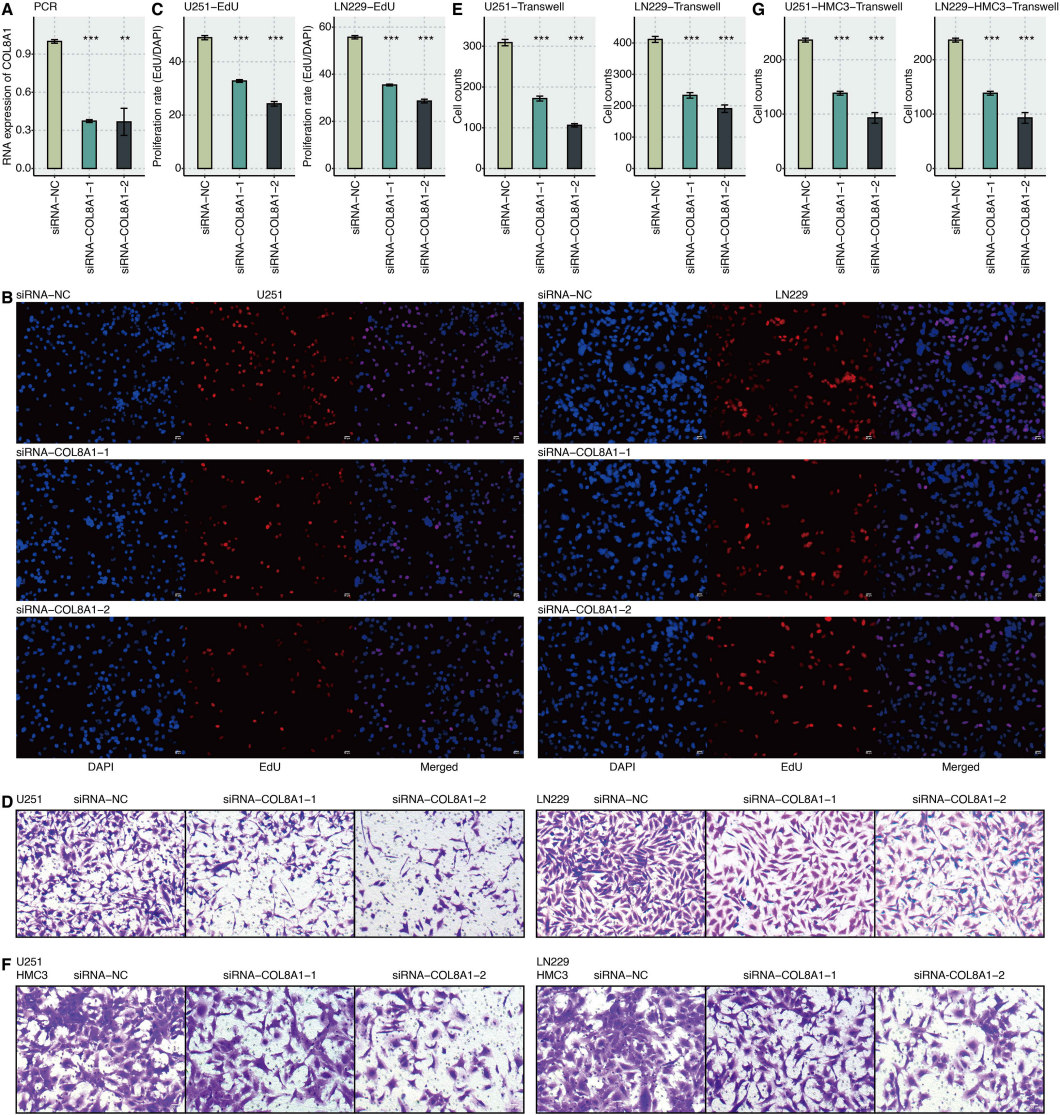
*In vitro* validation of COL8A1. **(A)** RT-qPCR assay shows the relative RNA expression of COL8A1 in normal control and two siRNA groups. **(B)** EdU assay shows the proliferated U251 and LN229 cells in the normal control and two siRNA groups. **(C)** Statistical analysis of EdU assay. **(D)** Transwell assay shows the migrated U251 and LN229 cells in the normal control and two siRNA groups. **(E)** Statistical analysis of the Transwell assay for U251 and LN229 cells. **(F)** Transwell assay shows the migrated HMC3 cells after coculture with U251 and LN229 cells in the normal control and two siRNA groups. **(G)** Statistical analysis of the Transwell assay for HMC3 cells. **P < 0.01; ***P < 0.001.

To experimentally validate the functional role of COL8A1 inferred from our bioinformatic analyses, we conducted a series of *in vitro* assays. We first designed two specific small interfering RNAs (siRNAs) to knock down COL8A1 expression in U251 and LN229 GBM cell lines. RT-qPCR confirmed a significant reduction of COL8A1 mRNA levels in both siRNA groups compared to the negative control (**Figure 5A**).

EdU incorporation assays demonstrated that silencing COL8A1 markedly suppressed the proliferative capacity of both U251 and LN229 cells (**Figures 5B, C**). Furthermore, Transwell migration assays revealed that COL8A1 knockdown significantly impaired the migratory ability of the GBM cells (**Figures 5D, E**). Given the importance of microglia-tumor cell interactions in GBM progression, we established a coculture system. We found that the conditioned medium from COL8A1-knockdown GBM cells significantly reduced the migratory capacity of HMC3 microglial cells compared to conditioned medium from control cells (**Figures 5F, G**). These results collectively demonstrate that COL8A1 intrinsically promotes GBM cell proliferation and migration and extrinsically modulates microglial behavior, contributing to a more aggressive tumor phenotype.

### COL8A1 sculpts an immunosuppressive tumor microenvironment and correlates with immune checkpoints

**Figure 6:**
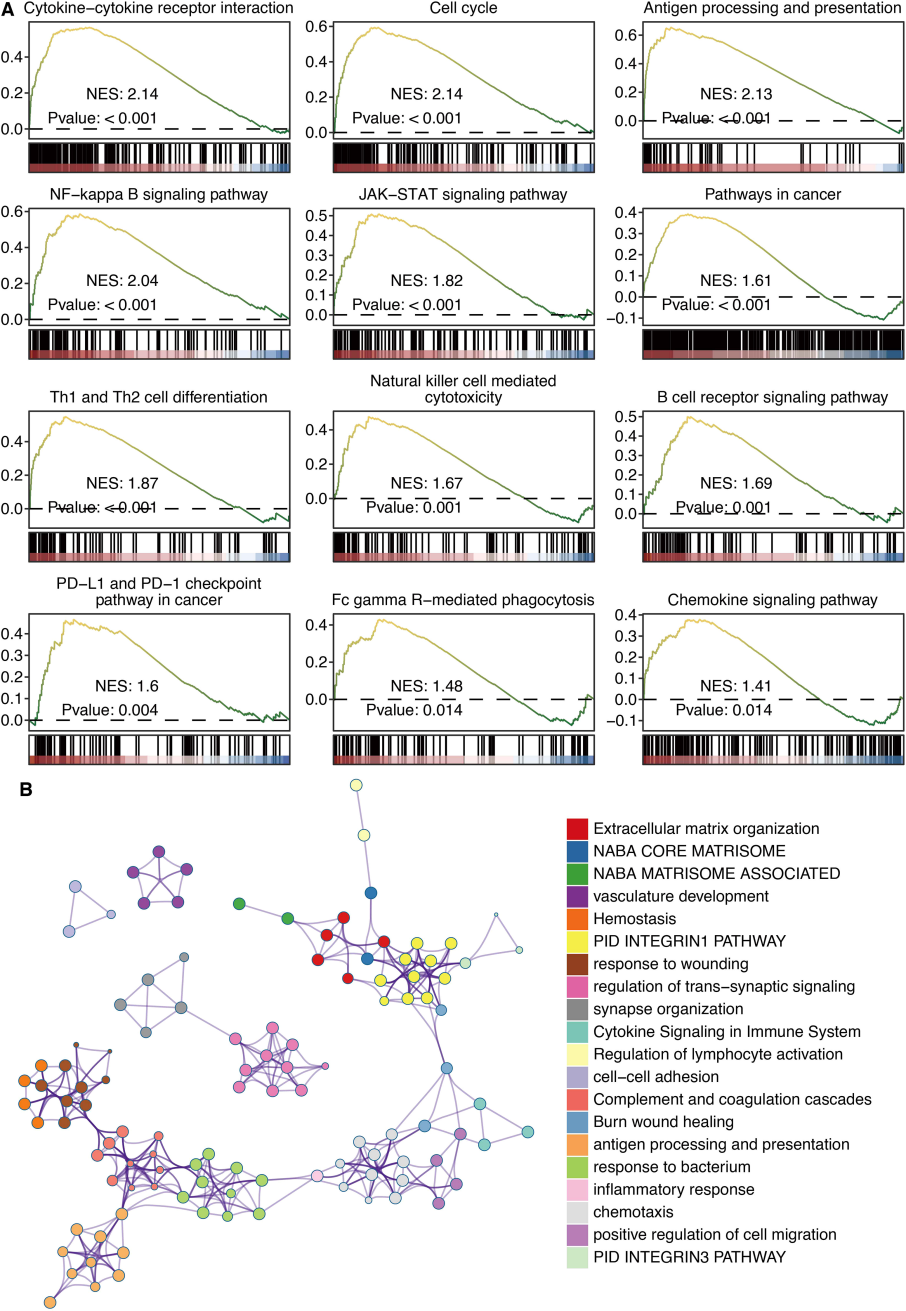
Immune functions of COL8A1 in glioma. **(A)** GSEA of KEGG pathways on COL8A1. **(B)** Pathway annotation of COL8A1 based on the Metascape platform.

To elucidate the mechanisms underlying COL8A1’s link to immunity, we performed GSEA of KEGG pathways. This analysis showed that high COL8A1 expression was significantly enriched in pathways related to B cell receptor signaling, T cell receptor signaling, natural killer cell-mediated cytotoxicity, and chemokine/cytokine activity (**Figure 6A**). Consistent with this, pathway annotation using the Metascape platform also highlighted “immune response” as one of the top biological processes associated with COL8A1 (**Figure 6B**).

**Figure 7:**
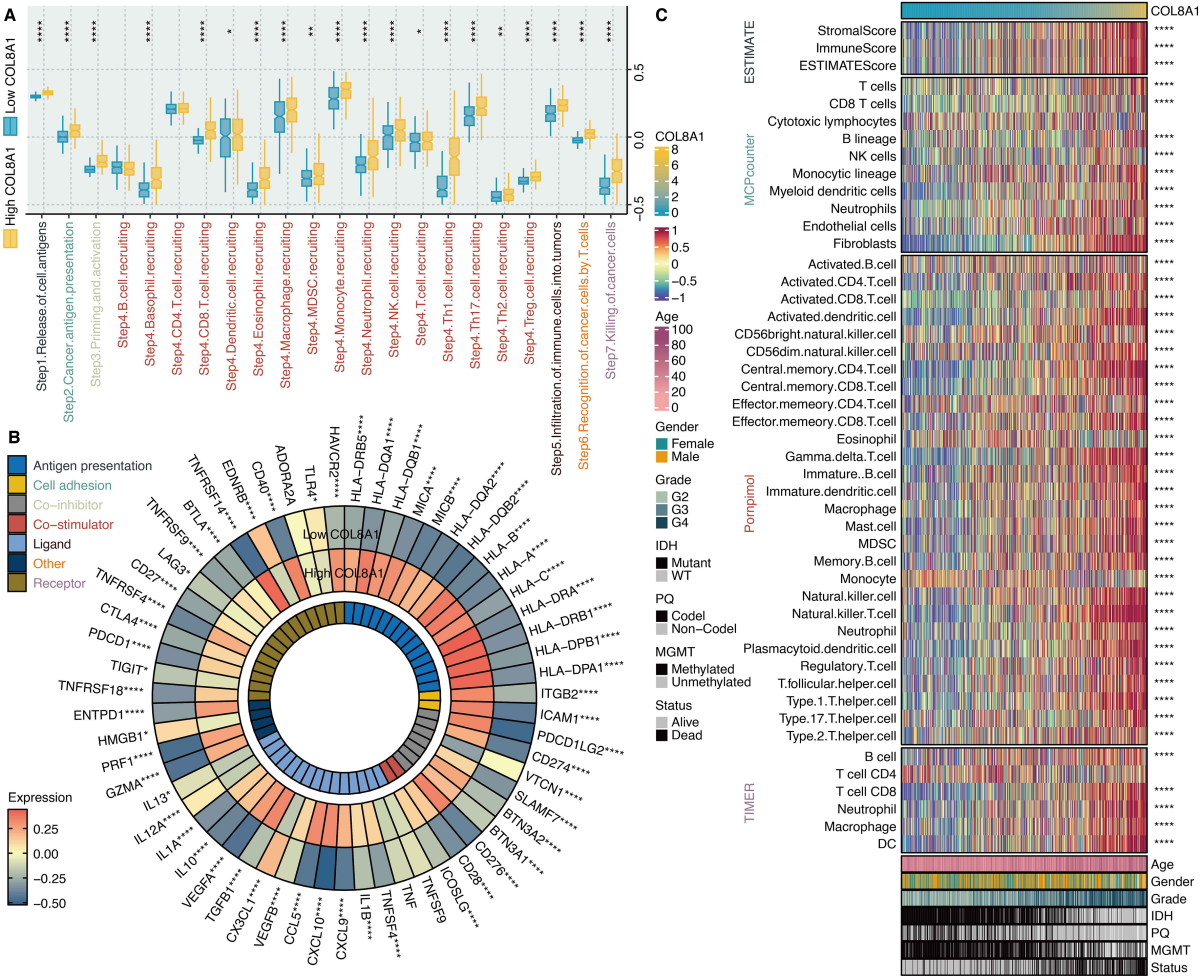
Immune features of COL8A1 in glioma. **(A)** The levels of immune cycles in COL8A1-based groups. **(B)** Heatmap shows the correlation between COL8A1 and classical immune modulators. **(C)** Heatmap shows the correlation between COL8A1 and ESTIMATE, MCP-counter, and Porpimol-based immune cells. *P < 0.05; **P < 0.01; ***P < 0.001.

We then quantitatively assessed the cancer immune cycle using a standardized framework. This analysis revealed that nearly all steps of the immune cycle, including immune cell recruiting, priming, activation, and trafficking, were significantly enhanced in the high COL8A1 group. Specifically, the recruitment of B cells, T cells, NK cells, Tregs, MDSCs, and macrophages was all elevated (**Figure 7A**). Crucially, despite the presence of a T-cell-inflamed phenotype, this immune-rich environment was coupled with immunosuppression. COL8A1 expression showed strong positive correlations with the expression of key inhibitory immune checkpoints such as PDCD1 (PD-1), CTLA4, and BTLA (**Figure 7B**). Comprehensive immune infiltration estimation using MCPcounter, ESTIMATE, and other algorithms confirmed that COL8A1-high tumors were characterized by significantly greater abundances of T cells, B cells, NK cells, and other immune populations (**Figure 7C**).

### COL8A1 as a predictive biomarker for immunotherapy response

**Figure 8:**
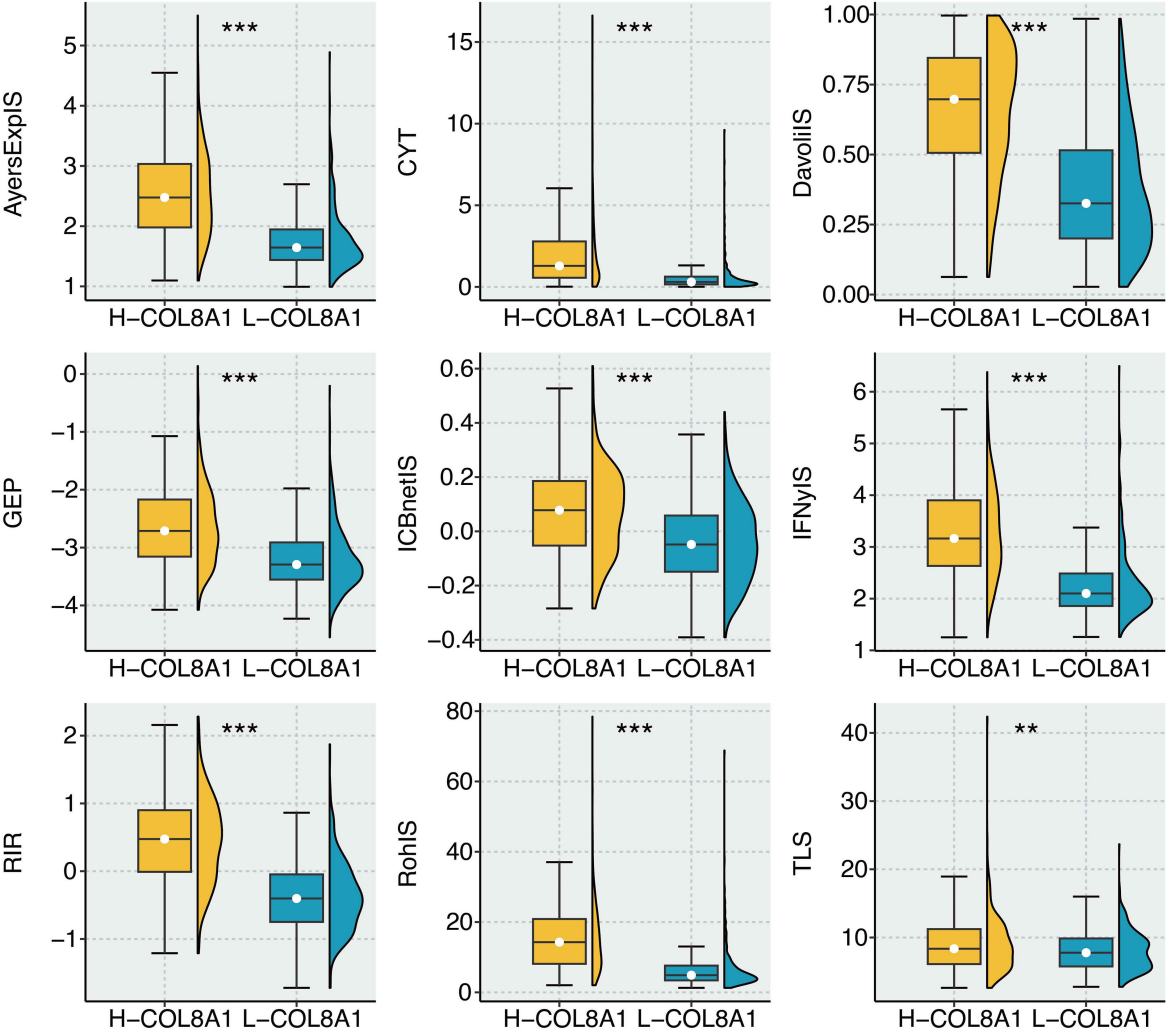
Immunotherapy prediction of COL8A1 in glioma. The scatter plot shows the correlation between COL8A1 and eight immunotherapy determinants. **P < 0.01; ***P < 0.001.

The presence of a T-cell-inflamed yet checkpoint-rich environment suggested that COL8A1 might predict response to immune checkpoint blockade (ICB). We evaluated the correlation between COL8A1 and nine well-validated immunotherapy determinants. COL8A1 was significantly positively correlated with all nine signatures, including CYT, FNγIS, ICBnetIS, AyersExpIS, GEP, RohIS, DavoliIS, a chemokineIS, and RIR signature (**Figure 8**).

**Figure 9:**
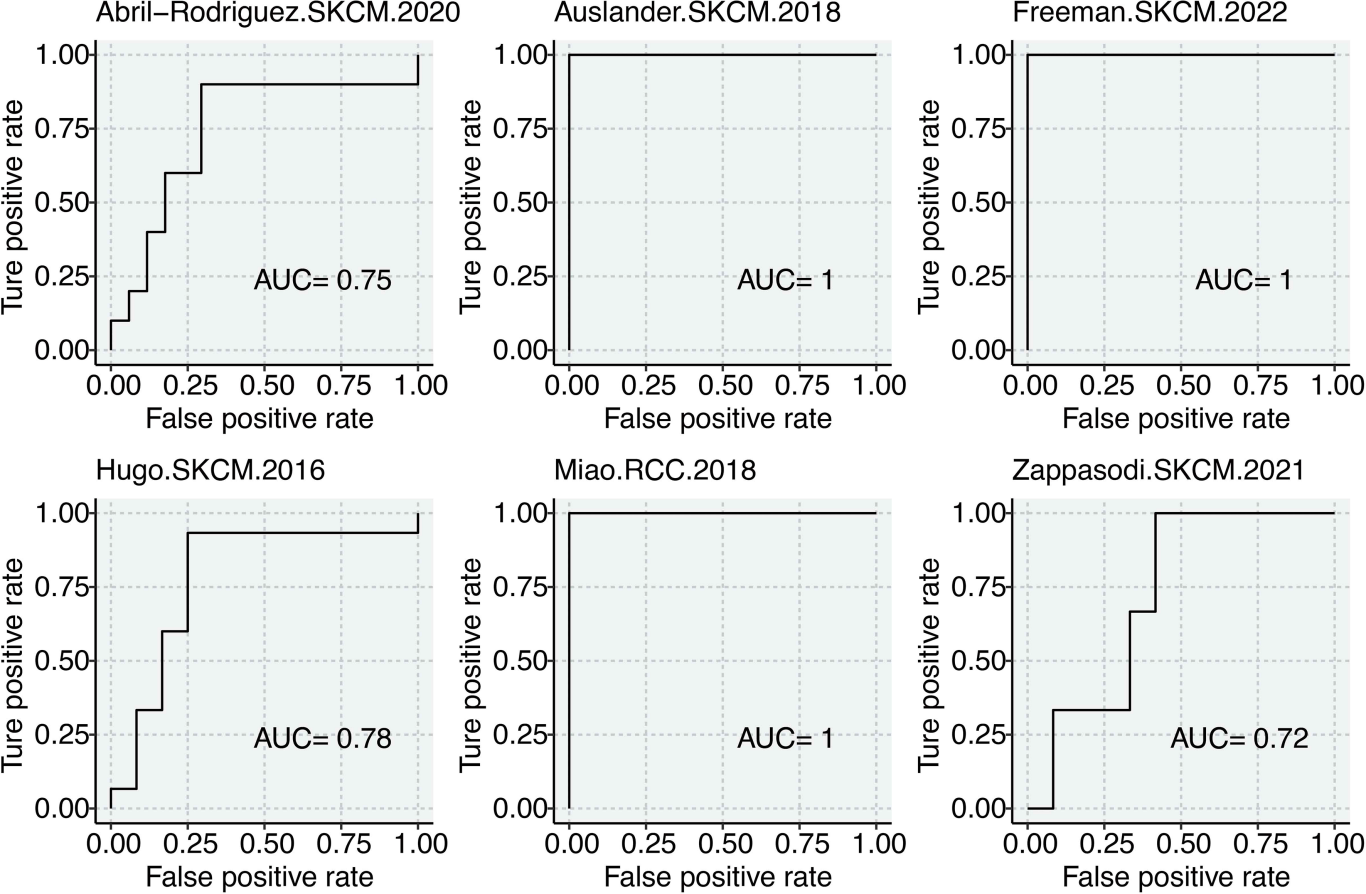
Immunotherapy prediction of COL8A1 in glioma. ROC curves show the efficacy of COL8A1 in predicting immunotherapy responses in six cohorts.

Most importantly, we directly assessed the predictive performance of COL8A1 using ROC analysis in six independent cohorts of patients who received ICB. The results demonstrated that COL8A1 expression alone could effectively distinguish between responders and non-responders, with AUC values consistently above 0.70 across multiple cohorts, indicating good predictive accuracy (**Figure 9**). This solidifies COL8A1’s potential utility as a novel biomarker for patient selection in glioma immunotherapy.

### Distinct somatic mutation landscapes are associated with COL8A1 expression

**Figure 10:**
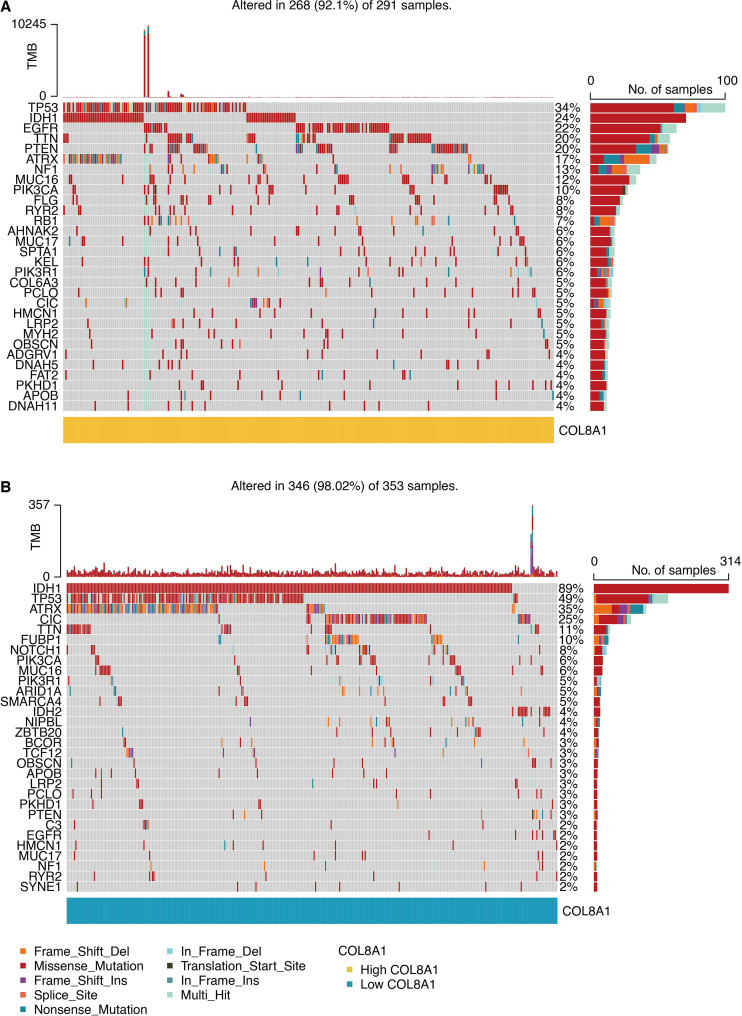
Mutation features of COL8A1 in glioma. **(A)** Waterfall plot shows the top-ranked mutated genes in glioma samples with high COL8A1 expression. **(B)** Waterfall plot shows the top-ranked mutated genes in glioma samples with low COL8A1 expression.

**Figure 11:**
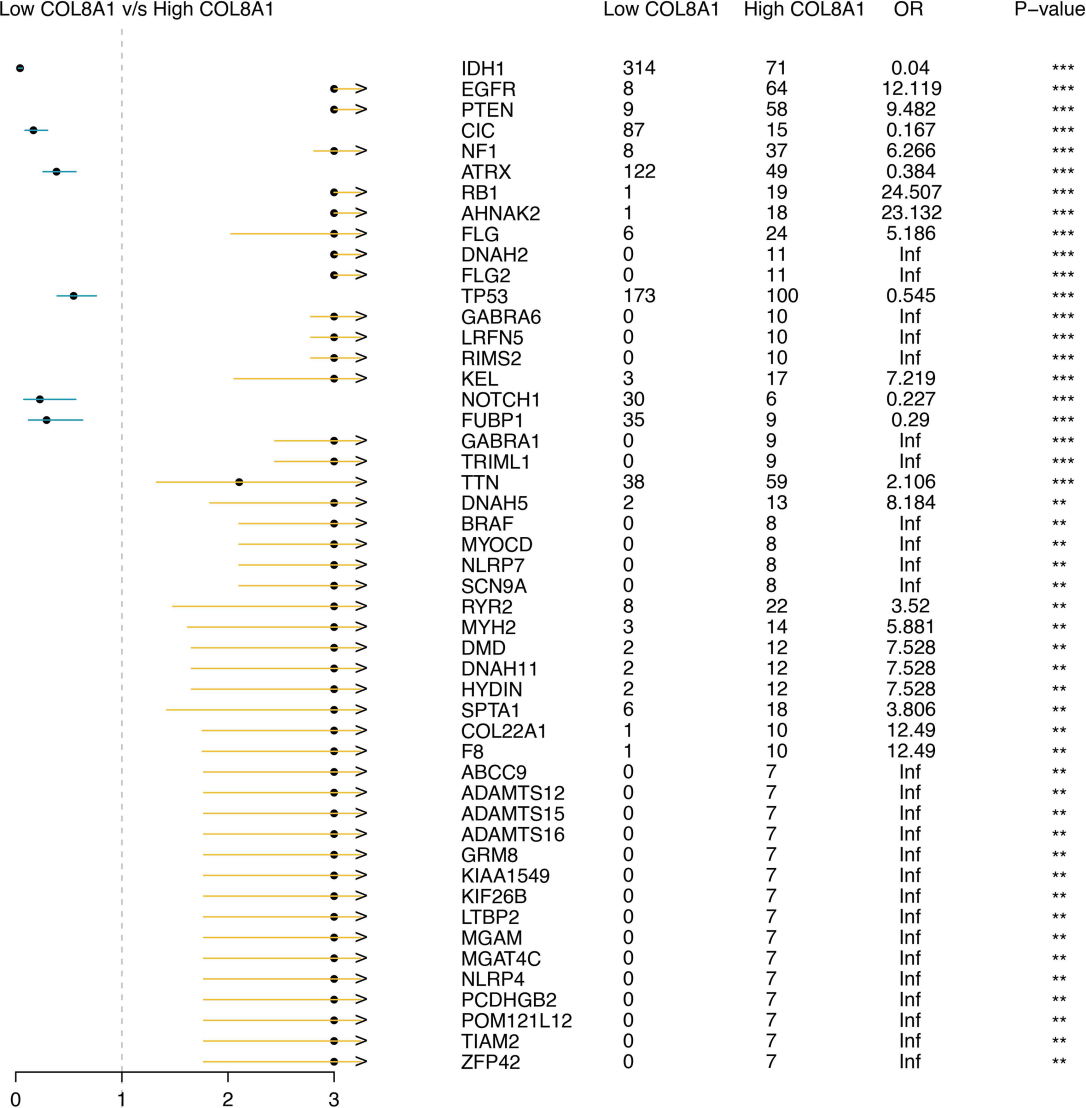
Mutation features of COL8A1 in glioma. The forest plot shows the differentially somatically mutated genes between COL8A1-based groups.

We next assessed the association between COL8A1 expression and genomic alterations in glioma. Analysis of the TCGA cohort revealed distinct mutational landscapes. The high COL8A1 group was dominated by oncogenic drivers, most frequently EGFR, which was often amplified and/or carried activating mutations (**Figure 10A**). In contrast, the low COL8A1 group was significantly enriched for mutations in the tumor suppressors IDH1 and TP53 (**Figure 10B**), alterations characteristic of the proneural subtype that often correlate with a more favorable prognosis. A forest plot of differentially mutated genes reinforced this dichotomy, with EGFR and PTEN alterations enriched in the high-expression group, and IDH1, CIC, and ATRX in the low-expression group (**Figure 11**). These findings position COL8A1 expression within specific, genetically defined glioma subtypes.

## Discussion

This study systematically identifies and validates COL8A1 as a pivotal inflammatory hub that bridges tumor-intrinsic properties, immune surveillance, and therapy response in glioma. Through an integrative multi-omics approach, we have moved beyond correlation to provide a mechanistic and clinical framework for understanding COL8A1’s role in this lethal disease.

Our study began with a single-cell resolution analysis of the inflammatory landscape in glioma. The identification of the blue module via hdWGCNA and its strong correlation with inflammation provided a high-confidence gene set from which COL8A1 emerged as the top prognostic candidate through a rigorous, multi-algorithm machine learning pipeline. Its consistent association with poor survival across nine independent cohorts, spanning both RNA-seq and microarray platforms, underscores its robustness as a prognostic biomarker, transcending technical and cohort-specific variations.

The functional significance of COL8A1 was confirmed by our *in vitro* experiments. The knockdown of COL8A1 significantly attenuated the proliferative and migratory capacities of GBM cells. This aligns with the known role of collagens in providing structural support and signaling cues that promote cell adhesion, survival, and motility (46). More importantly, our coculture experiment revealed a novel paracrine function of COL8A1: its expression in tumor cells enhances the migration of microglia. This suggests that COL8A1 is not only a tumor-intrinsic aggressiveness factor but also an active modulator of the TME, potentially facilitating the recruitment of pro-tumorigenic microglia/macrophages to the tumor site, which in turn can foster angiogenesis, invasion, and immunosuppression (47).

Perhaps the most intriguing finding of our study is the dual immune-modulatory role of COL8A1. On one hand, high COL8A1 expression is associated with a robust T-cell-inflamed phenotype, characterized by the enrichment of immune cell recruiting signals and the elevated presence of T, B, and NK cells (48, 49). This “hot” immune contexture is generally considered a prerequisite for response to ICB. On the other hand, this inflamed state is coupled with strong immunosuppressive signals, including the upregulation of multiple inhibitory checkpoints (PD-1, CTLA-4, BTLA) and the recruitment of Tregs and MDSCs. This creates a paradoxical state of “immune-rich suppression,” where anti-tumor immune cells are present but are functionally restrained. This duality likely explains the aggressive nature of COL8A1-high tumors while also providing a rationale for their potential susceptibility to ICB. Our analysis confirms this by demonstrating that COL8A1 is a strong predictor of positive response to immunotherapy across multiple cohorts. It acts as a composite biomarker, capturing both the necessary inflamed context and the targetable immunosuppressive mechanisms.

The mutational landscape analysis further contextualizes COL8A1 within glioma molecular subtypes. The strong association of high COL8A1 with EGFR mutations (a hallmark of the classical subtype) and low COL8A1 with IDH1/TP53 mutations (characteristic of the proneural subtype) suggests that COL8A1 is a key feature of the more aggressive classical/mesenchymal subtypes. This integration of transcriptomic, functional, and genomic data provides a more holistic view of glioma stratification.

Several limitations of this study should be acknowledged. While our *in vitro* data support a functional role for COL8A1, *in vivo* studies using animal models are required to fully validate its impact on tumor growth, TME remodeling, and response to therapy in a more physiologically relevant context. The precise molecular mechanisms by which COL8A1, presumably an extracellular matrix protein, regulates intracellular signaling in tumor cells and communicates with immune cells remain to be elucidated. It may interact with integrin receptors or modulate the availability of growth factors and cytokines in the TME. Furthermore, the predictive value of COL8A1 for immunotherapy, while promising, requires prospective validation in clinical trials.

## Conclusion

In conclusion, our work unveils COL8A1 as a central node in a pathogenic network that drives glioma aggressiveness by synchronizing tumor-intrinsic inflammation with the sculpting of an immunosuppressive TME. It functions as a potent prognostic indicator, a promoter of tumor cell proliferation and migration, a regulator of immune cell recruitment and checkpoint expression, and a predictive biomarker for immunotherapy response. These multifaceted roles nominate COL8A1 not only as a valuable clinical tool for patient stratification but also as a compelling therapeutic target. It is important to acknowledge the limitations of this study. While our bioinformatic discoveries and *in vitro* validations provide a strong foundation, the functional role of COL8A1 and its efficacy as a therapeutic target remain to be fully validated *in vivo* using animal models. Furthermore, the precise molecular mechanisms, specifically, the receptors through which COL8A1 signals and the detailed downstream pathways it activates in both tumor and immune cells, are yet to be elucidated. Finally, the promising predictive value of COL8A1 for immunotherapy response, while robust across multiple retrospective cohorts, requires prospective validation in clinical trials.

## Data Availability

The original contributions presented in the study are included in the article/**Supplementary Material**. Further inquiries can be directed to the corresponding authors.

## Supplementary material

Supplementary Figure 1 **(A)** Intersected genes among hdWGCNA blue module genes, upregulated genes in tumor cells, and DEGs between -based clusters. **(B)** The expression of COL8A1 in microenvironment cells.

## Glossary

AUC: Area Under the Curve
BTLA: B and T Lymphocyte Attenuator
CGGA: Chinese Glioma Genome Atlas
COL8A1: Collagen Type VIII Alpha 1 Chain
CTLA-4: Cytotoxic T-Lymphocyte-Associated Protein 4
CYT: Cytolytic Activity
DC: Dendritic Cell
DEG: Differentially Expressed Gene
ECM: Extracellular Matrix
EdU: 5-Ethynyl-2’-deoxyuridine
EGF: Epidermal Growth Factor
EGFR: Epidermal Growth Factor Receptor
FPKM: Fragments Per Kilobase of transcript per Million mapped reads
GBM: Glioblastoma
GEP: Gene Expression Profile
GSEA: Gene Set Enrichment Analysis
hdWGCNA: high-dimensional Weighted Gene Co-expression Network Analysis
HALLMARK: MSigDB Hallmark Gene Sets
ICB: Immune Checkpoint Blockade
ICBnetIS: Immune Checkpoint Blockade Network Immunophenotype Score
IDH1: Isocitrate Dehydrogenase 1
IFNγIS: Interferon Gamma Immunophenotype Score
KEGG: Kyoto Encyclopedia of Genes and Genomes
LASSO: Least Absolute Shrinkage and Selection Operator
MDSC: Myeloid-Derived Suppressor Cell
NF-κB: Nuclear Factor Kappa-Light-Chain-Enhancer of Activated B Cells
NK Cell: Natural Killer Cell
OPC: Oligodendrocyte Precursor Cell
PAM: Partitioning Around Medoids
PCA: Principal Component Analysis
PD-1: Programmed Cell Death Protein 1
PDCD1: Programmed Cell Death 1 (gene for PD-1)
RIR: Root Immune Response
ROC: Receiver Operating Characteristic
RMA: Robust Multi-array Average
RT-qPCR: Quantitative Reverse Transcription Polymerase Chain Reaction
scRNA-seq: Single-Cell RNA Sequencing
siRNA: Small Interfering RNA
TCGA: The Cancer Genome Atlas
TME: Tumor Microenvironment
TP53: Tumor Protein P53
TPM: Transcripts Per Million
Treg: Regulatory T Cell
UMAP: Uniform Manifold Approximation and Projection.
