# Supplementary material for: COL8A1 as a pro-inflammatory mediator bridges immune evasion and therapy resistance in glioma
Source: Front Immunol. 2025 Dec 10;16:1727298. doi: 10.3389/fimmu.2025.1727298 (PMC12727913; doi:10.3389/fimmu.2025.1727298)
Supplement: Supplementary Figure 1 — (A) Intersected genes among hdWGCNA blue module genes, upregulated genes in tumor cells, and DEGs between -based clusters. (B) The expression of COL8A1 in microenvironment cells. [file DataSheet1.docx]

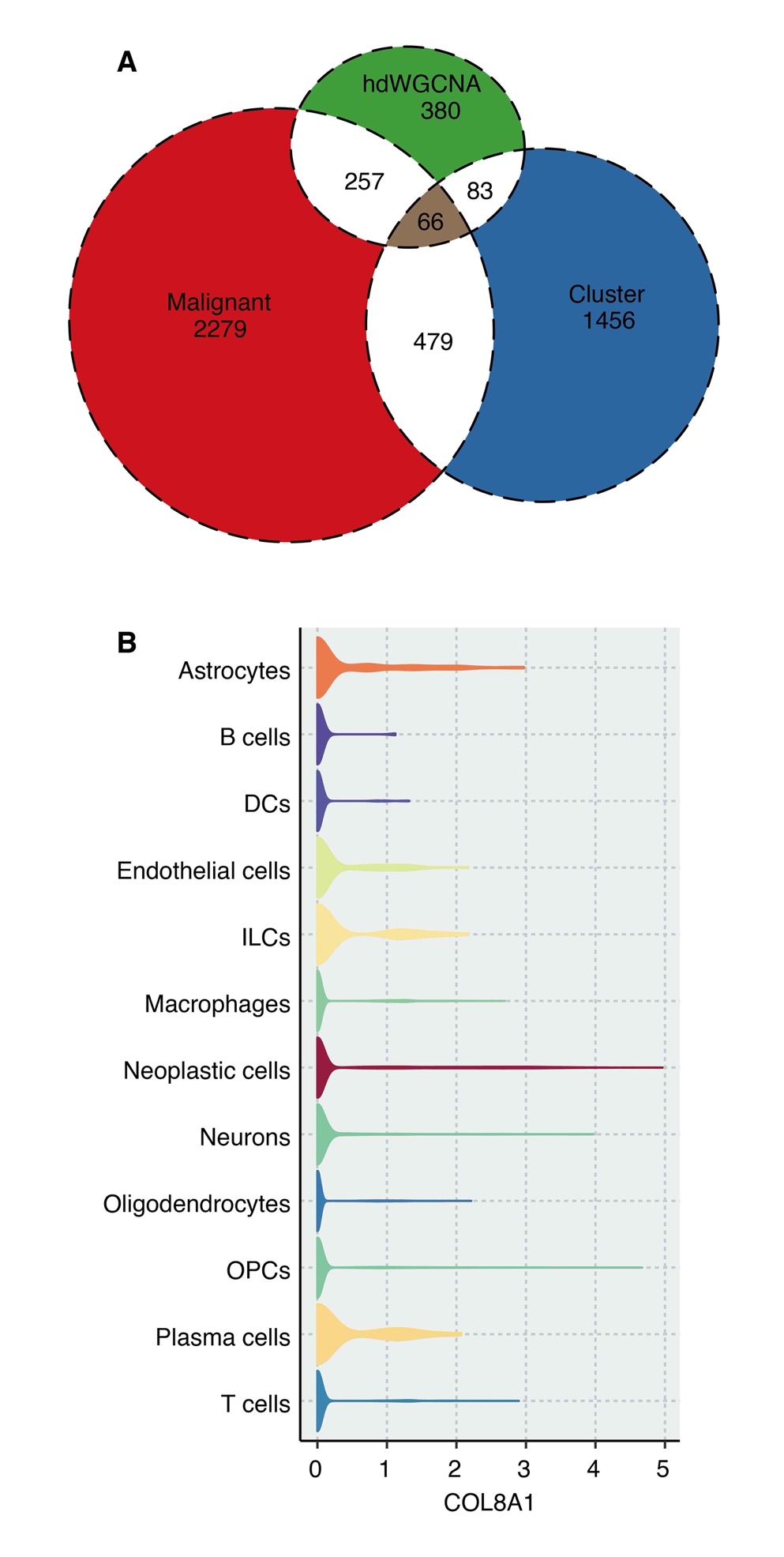


Figure S1. A. Intersected genes among hdWGCNA blue module genes, upregulated genes in tumor cells, and DEGs between -based clusters. B. The expression of COL8A1 in microenvironment cells.
